# From numerical to empathy: the dual impact of psychological contracts in doctor-patient communication

**DOI:** 10.3389/fpsyt.2025.1530932

**Published:** 2025-10-24

**Authors:** Xinru Wang, Yating Chen, Yi Yu, Huan Jiang, Jinyan Song, Weixian Liang, Qiang Zhou, Liang Ying

**Affiliations:** 1 Wenzhou Medical University, Wenzhou, China; 2 Soochow University, Suzhou, China

**Keywords:** pain, empathy, psychological contracts, doctor-patient relationship, probability estimation bias

## Abstract

**Objective:**

To investigate how the presence or absence of psychological contracts and different formats of probabilistic data representation influence healthcare professionals’ pain empathy and probability estimation bias in simulated doctor–patient communication contexts.

**Methods:**

We included 60 healthcare professionals with the same mathematical ability and divided them into two groups to complete the probability estimation bias task of decision events and the classification task of pain non-pain pictures with and without psychological contracts. The data are analyzed by generalized estimation equation (Gee).

**Results:**

The fulfillment of psychological contracts significantly affects the level of empathy for pain[0.3(95% CI 0.1, 0.4), *p <*0.001], and the probability bias of decision events with an impact of [19.2 (95% CI 8.5, 29.8), *p <*0.001] in small probability events and [−21.2 (95% CI −41.7, −0.5), *p*<0.05] in large probability events.

**Conclusions:**

The establishment of psychological contract reduced the difference between the different data representation forms, significantly improved the pain empathy of the healthcare professionals, and reduced the probability estimation bias of risk decision events.

## Introduction

A harmonious doctor-patient relationship is essential for the smooth progression of medical activities. However, the relationship between doctors and patients is becoming increasingly strained, with a significant rise in disputes (1). Consequently, mediating these conflicts and enhancing doctor-patient communication have become a focal point for all sectors of society. The understanding of doctor-patient relationships has transitioned from one-way authority to two-way interaction. Currently, the Shared Decision-Making (SDM) model enhances treatment adherence through the joint participation of doctors and patients in decision-making (2); while recent studies further emphasize the partnership theory, viewing patients as equal members of the healthcare team (3). To realize such collaboration and partnership in medical practice, effective communication and understanding are key, with empathy playing a central role.

Empathy involves understanding a patient’s situation, perspective, and emotions; effectively communicating this understanding to the patient; and using this insight to provide therapeutic benefits (4). In the early days, compassion was mainly seen as an emotional response and care of physicians towards the suffering of patients (5). As empirical research progressed, the role of compassion was revealed more comprehensively. High levels of physician compassion have been proven to significantly improve patient satisfaction, treatment compliance, and clinical outcomes (6, 7). Notably, physicians’ effective communication of empathy during interactions can significantly predict the quality of the doctor-patient relationship, and this effect mainly operates through improving doctor-patient communication (8).

Most discussions on doctor-patient communication from the perspective of empathy are primarily theoretical. Numerous studies have explored empathy skills and doctor-patient communication from a humanistic perspective (9), while few have considered this topic from an empirical research standpoint.

In addition, empathy is redefined as a learnable psychological skill (10), rather than merely an innate emotional expression. In specific fields such as pain management, the importance of pain empathy is increasingly highlighted and considered a core element that impacts patient experience and treatment outcomes (3). Pain empathy refers to the perception and emotional response one has to another person’s suffering (11), influenced by situational (12). Psychological contracts play a crucial role in the doctor-patient context. These contracts refer to an implicit understanding and trust between patients and doctors, beyond formal agreements. For patients, this means expecting professional, compassionate, and respectful care from doctors. Conversely, doctors expect patients to follow their medical advice and treatment plans. Research has indicated that breaches in psychological contracts can result in antisocial behaviours among patients (13). Furthermore, the level of empathy for pain largely depends on the probability of the stimulus (another person’s pain). The probability of an event reflects the expected likelihood of its occurrence (14). Research has shown that the anticipation of pain, even before it occurs, influences an individuals’ pain perception (15). Therefore, understanding how physicians assess and communicate the likelihood of pain occurrence is crucial for building patient trust.

When examining doctor-patient relationships, empathy and psychological contracts are pivotal concepts that can significantly enhance the quality of communication and foster greater patient trust in physicians. However, beyond empathy and psychological contracts, the way patients perceive and understand risk and treatment options also plays a critical role in shaping doctor-patient interactions. Probability estimation bias in decision-making events refers to the discrepancy between the subjective assessment of the likelihood of an event and its objective probability. Medium-to-high probabilities tend to be underestimated, whereas low probabilities are often overestimated (16, 17). Probability estimation bias is influenced by mathematical perception ability and data presentation format (18, 19). The higher people’s mathematical perceptual ability and the more thorough their understanding of probabilistic information, the less biased the probability estimates of decision events.^16^ In addition, risk information is easier to understand when presented in the form of frequencies rather than probabilities. For example, discrete frequency formats such as “X-in-100” (meaning there is a probability of X parts in a whole of one hundred) or “1-in-X” (meaning there is a probability of X parts in a whole of one unit) are often preferred to percentage or probability formats (20). This bias in doctor-patient communication may lead to misinterpretation or exaggeration of risks, causing patients to have unrealistic expectations or concerns about treatment outcomes, thereby affecting their trust in doctors and treatment adherence. Therefore, when discussing how psychological contracts and empathy influence doctor-patient relationships, it is necessary to consider probability estimation bias, as it plays an essential role in patients’ perception of risk, understanding of treatment options, and decision-making processes. Only by integrating research on empathy, psychological contracts, and probability estimation bias can we gain a more comprehensive understanding of the mechanisms that influence doctor-patient communication and trust.

To improve healthcare professionals’ decision-making accuracy in the workplace and their empathy in patient-physician communication, we asked the following research questions: What forms of data representation can reduce bias in probability estimation in risk assessment, and improve empathy in patient-physician communication among healthcare professionals? Can psychological contracts enhance health care professionals’ empathy and amplify the impact of data representation forms? By examining these questions, we expect to determine the optimal form of data representation for improving the quality of healthcare services. Accordingly, this study focuses on healthcare professionals and examines how psychological contracts (present vs. absent) and data representation formats (1-in-X, X-in-100, X%) influence pain empathy and probability estimation bias. The study employs laboratory-based experiments and computer tasks to simulate doctor–patient interactions. Through this design, the study aims to clarify how psychological contracts and different data presentation formats shape physicians’ cognitive and emotional responses.

## Materials and method

### Participants

Sixty healthcare professionals participated in this study. Among them, 46 healthcare professionals aged 25–34 years old (M=29.42 years, 11 males), 11 healthcare workers aged 35–44 years old (M=38 years, 4 males) and 3 healthcare workers aged 45–54 years old (M=51.34 years, 1 male). Before the beginning of the experiment, the participants’ math level was examined with a questionnaire, and all the participants answered all the questions about calculations correctly, and the participants were at the same level of math.

### Measure

#### Mathematical ability questionnaire

The mathematical ability questionnaire developed by Schwartz et al. to assess participants’ numerical ability (21), which consists of a total of eleven questions, was applied to a measure of math level ability prior to probability estimation. This questionnaire has been widely used in several studies to ensure that all participants have a similar mathematical foundation when performing probability estimation tasks, thereby controlling for potential impacts of differences in mathematical ability on the research outcomes (22–24).

#### Probability estimation bias of decision events

Adapted from the bias paradigm for probability estimation bias of decision events developed by Tversky and Kahneman (25), the paradigm consists of 22 decision questions, each containing two choices. Among them, option A is the risky option (there is a probability of pi to get Xi dollars and a probability of (1-pi) to get Yi dollars), and option B is the certainty option (100% to get ______dollars). The participant’s experimental task was to fill in the space for option B with a determined subjective perception value such that it was the same as the subjective perceptibility of option A. In the experiment, the probabilities in the titles were expressed in three formats: 1-in-X, X-in-100, and X%. Twenty-two probability groups were randomly selected, with eleven groups representing high-probability events and eleven groups representing low-probability events.

### Procedure

This experiment is divided into three parts: questionnaire collection, scenario introduction and computer experiment. The first was the questionnaire collection section, in which participants were given a mathematical ability questionnaire to measure their level of math. The second part was the introduction of the scenario, which controlled for the presence or absence of the psychological contract situation. In the psychological contract fulfillment scenario, participants were informed in advance that they would be given a b gift before the experiment to establish the contract; before the experiment officially started, participants were given a gift to fulfill their promise; after the experiment, a brief survey was conducted with the doctors. The survey aimed to assess whether they perceived the fulfillment of the promised gift and whether it elicited positive emotions. In the no-contract scenario, the procedure was as follows: no gift was promised, and after the experiment, a gift was given to thank the participants for their participation.

In this study, the gift is defined solely as a “trigger” used to activate the psychological contract, rather than being the core component of the contract itself. Specifically, by informing doctors in advance about receiving a gift, the formation of the psychological contract is activated. This clear expectation may enhance doctors’ perception of their own responsibility, thus improving their behavioral performance (26). The gifts used in the experiment were medical-themed souvenirs, selected to emphasize their symbolic significance rather than material value. They served solely as a neutral medium to embody the “promise–fulfillment” process, while avoiding potential confounds or misinterpretations associated with material incentives. In this way, the gift functioned only as a “trigger” within the study, facilitating the activation of the psychological contract. Moreover, these souvenirs help avoid the potential drawbacks associated with material exchanges, further emphasizing the emotional aspect of the gesture. This approach aligns with the conceptualization of the gift as a “trigger” for initiating the psychological contract.

**Figure 1:**
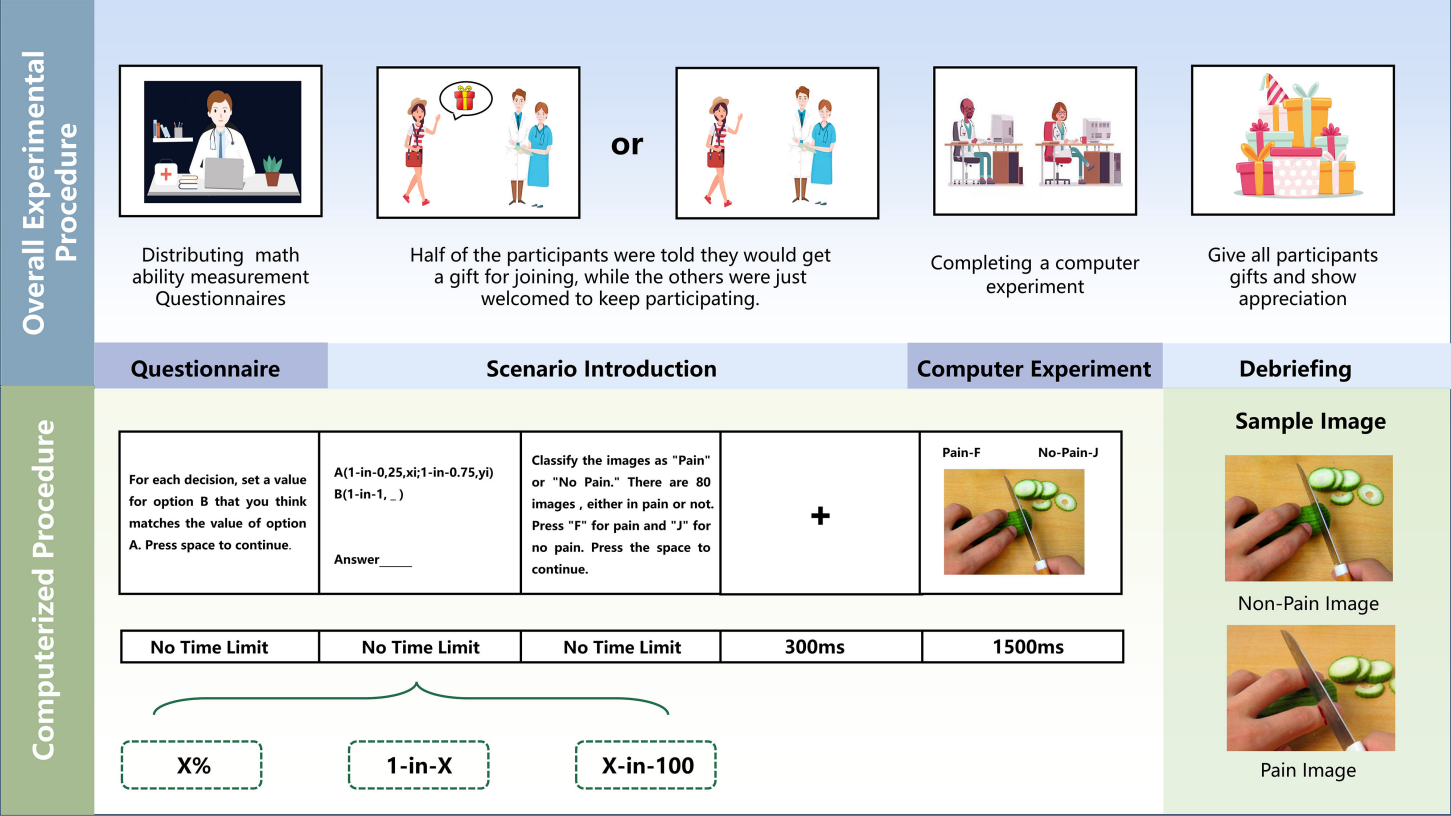
Overall experimental procedure and computerized procedure.

The final experiment used PsychoPy 3 and consisted of two parts: measuring probability estimation bias in decision making and measuring pain empathy. Participants completed 22 decision-making tasks for the probability estimation bias measurement. For the pain empathy measurement, they classified 80 image stimuli as painful or non-painful, and their accuracy and reaction times were recorded. The experimental procedure is shown in **Figure 1**.

### Data analysis

Statistical analyses were performed with the Generalized Estimating Equations (GEE) with SPSS, this study examines the variations in Variable 1 (decision event probability estimation bias) and Variable 2 (pain empathy level) under different conditions (presence/absence of psychological contract fulfillment), methods (different numerical representation forms: 1-in-X, X-in-100, X%), and types (large/small probability events, pain/non-pain images), as shown in **Table 1** Results of Generalized Estimating Equations (GEE) analysis. To further explore the differences in estimation bias and pain empathy levels across psychological contract fulfillment, numerical representation forms, and large/small probability events, pairwise comparisons were conducted. In all cases, only significant determinants (*p*<0.05) were included in the final model to ensure an accurate and effective analysis of the determinants. Since there was no significant difference in response accuracy between the two groups (*p* > 0.05), only response time was analyzed.

**Table 1:** Results of Generalized Estimating Equations (GEE) analysis (**p<.01, ***p<.001).

Random effects	Overestimation of small probability bias		Underestimation of probability bias		Response times probability estimation deviation	
	Estimate(S_R_)		*P* value		Estimate(S_R_)	*P* value
Subjects(intercept)	45.47(8.98)		<0.001***		1.10(0.31)	<0.001***
Fixed Effects	F(df)	*P* Value	F(df)	*P* Value	F(df)	*P* Value
Psychological contract	87.00(1,54)	<0.05**	58.95(1,54)	<0.05**	16.00(1,54)	<0.05**
Numerical manifestations	14.59(2,54)	<0.05**	13.12(1,54)	<0.05**	6.07(2,54)	<0.05**
Psychological contract × Numerical manifestations	5.17(2,54)	<0.05**	2.74(2,54)	<0.05**	0.33(2,54)	0.727

## Result

### Probability estimation bias

#### Psychological contract

**Figure 2:**
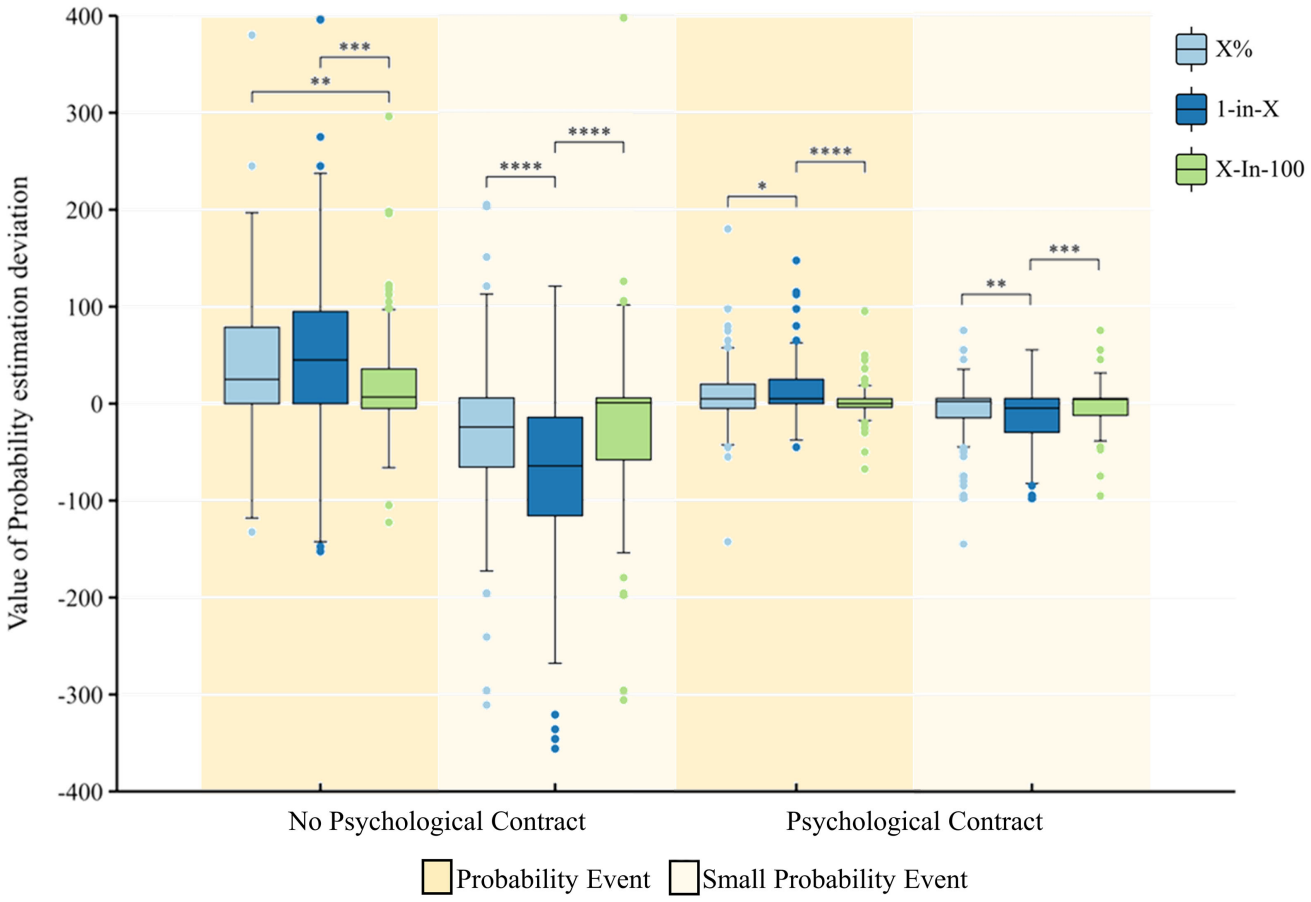
Estimated bias values for the probability of decision events with and without psychological contract fulfilment and with different manifestations. *p < .05, **p < .01, ***p < .001, ****p < .0001. All coefficients are standardized.

There was a significant difference in the effect of psychological contract fulfillment on the probability estimation bias of decision events, whether in small probability events [19.2 (95% CI 8.5, 29.8), *p*<0.001] or in large probability events [−21.2 (95% CI −41.7, −0.5), *p*<0.05] (**Figure 2**). In small probability events, the interaction between psychological contract fulfilment and “X%” was significant [38.6(95% CI 20.1, 57.1), *p*<0.001];the interaction between psychological contract fulfilment and “X−in−100” was significant, both in small probability events [40.7(95% CI 4.1, 77.3), *p*<0.05] and large probability events [−54.8(95% CI −86.3, −23.4), *p*<0.001]; and the interaction of psychological contract fulfilment and “1−in−X” was significant. Both in small probability events [19.2 (95% CI 8.5, 29.8), *p*<0.001] and large probability events [−21.2 (95% CI −41.8, −0.5), *p*<0.05].

#### Data presentation format

There was a significant difference in the effect of “1−in−X “on the probability estimation bias of decision events, whether in small probability events [12.9 (95% CI 9.3, 16.7), *p*<0.001] or in large probability events [−13.7 (95% CI −26.1, −1.4), *p*<0.05] (**Figure 2**). In small probability events, the “X%” group is significantly more highly valued than the “X−in−100” group [13.8(95% CI 3.1, 24.5), *p*<0.05], and the “1−in−X “group is also significantly more highly valued than the “X−in−100” group [23.7(95% CI 4.7, 42.8), *p*<0.05]. In the probability event, the “1-in-X” group undervalues it significantly more than the “X%” group [29.5(95% CI 9.2, 49.7), *p*<0.01] and the “X-in-100 “ group [−30.6(95% CI −49.2, −11.8), *p*<0.01].

### The level of empathy for pain

#### Psychological contract

**Figure 3:**
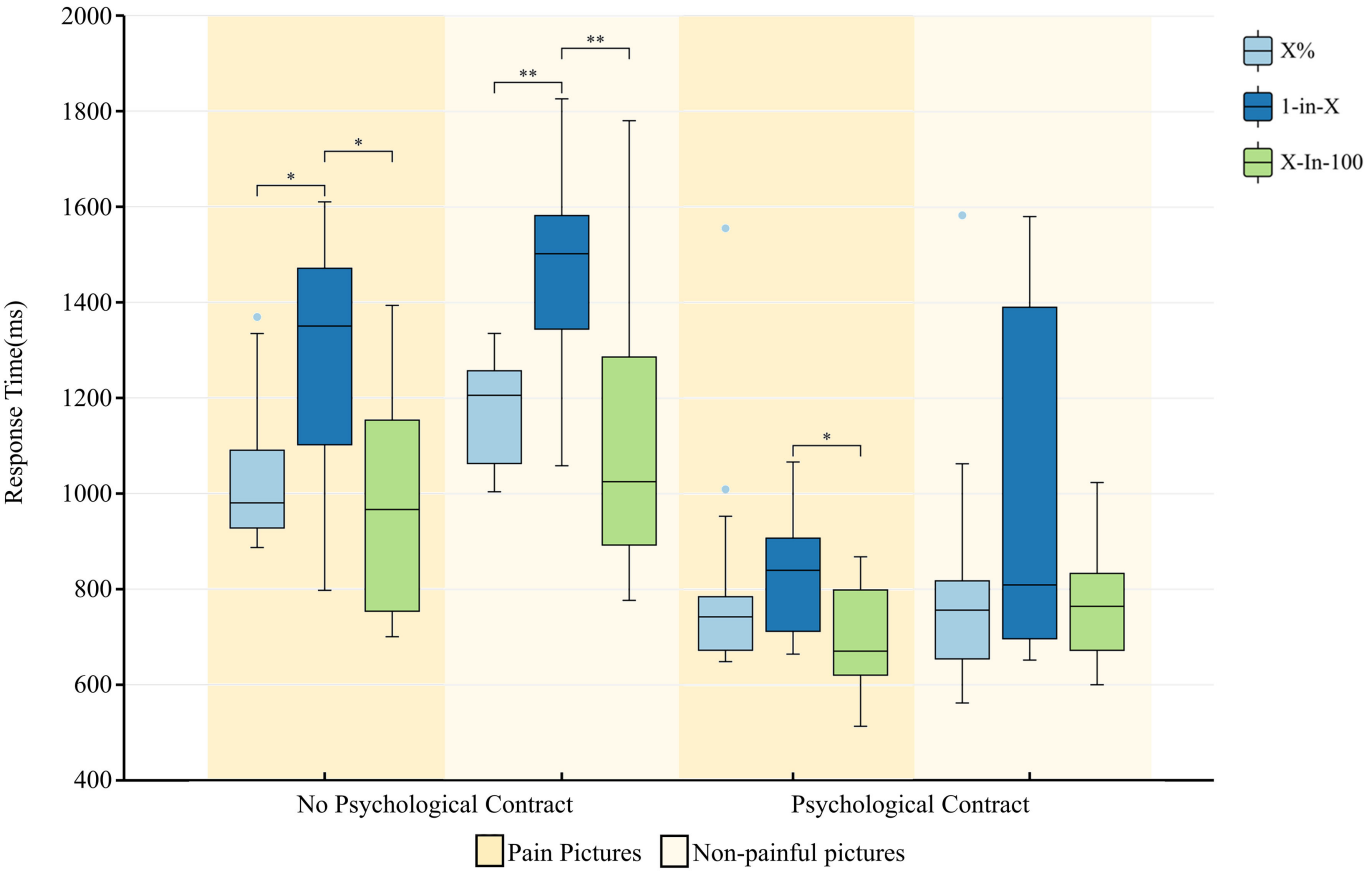
Presence or absence of psychological contract fulfilment and level of pain empathy in different manifestations. *p < .05, **p < .01. All coefficients are standardized.

There was a significant difference in the effect of psychological contract fulfilment on the level of pain empathy, both in pain pictures [0.3(95% CI 0.1, 0.4), *p*<0.001] and in non−pain pictures [0.3(95% CI 0.1, 0.5), *p*<0.001] (**Figure 3**). The interaction between psychological contract fulfilment and “X%” was significant, both in pain pictures [0.2(95% CI 0.02, 0.4), *p*<0.05] and in non−pain pictures [0.3(95% CI 0.2, 0.5), *p*<0.001]; the interaction between psychological contract fulfilment and “1−in−X” interaction was significant, both in pain pictures [0.3(95% CI 0.04, 0.6), *p*<0.05] and in non−pain pictures [0.6(95% CI 0.3, 0.9), *p*<0.001]; the interaction between psychological contract fulfilment and “X−in−100” interaction was significant, both in pain pictures [0.3(95% CI 0.1, 0.5), *p*<0.001] and in non−pain pictures [0.3(95% CI 0.1, 0.5), *p*<0.01].

#### Data presentation format

Here was a significant difference in the effect of “1−in−X” on the level of pain empathy [0.3(95% CI 0.01, 0.5), *p*<0.05] (**Figure 3**). In the pain pictures, the “1−in−X” group had a longer response time than the “X%” group [−0.2(95% CI −0.4, −0.01), *p*<0.05] and the “X−in−100 “ group [0.3(95% CI 0.1, 0.4), *p*<0.001]; and in the non−pain picture, the “1−in−X” group took longer to respond than the “X%” group [−0.3(95% CI −0.4, 0.1), *p*<0.05] and “X−in−100” group [0.3(95% CI 0.2, 0.5), *p*<0.001].

## Discussion and conclusion

### Discussion

This study explored the impact of psychological contracts and data presentation formats on probability estimation bias and pain empathy from healthcare professionals’ perspectives. It investigates which psychological contract fulfillment and data presentation formats can reduce probability estimation bias and enhance the level of pain empathy among healthcare professionals.

First, for probability estimation bias, it was concluded that in the absence of a mental contract, the probability estimation bias under the discrete frequency format X-in-100 is significantly smaller than that under the other two formats, which means that the probability can be estimated more accurately in the frequency display than in the percentage display. It was found that people assessed risk information more accurately when the data were presented in a way that conformed to people’s mental models (27), implying that the numerical presentation of X-in-100 was easier to communicate and understand and more in line with people’s mental models. Under the condition of psychological contract establishment, the probability estimation bias under each numerical format was significantly reduced; however, the probability estimation bias under the discrete frequency format X-in-100 was still the smallest under both large and small probability events.

Hoffrage et al. provided additional support for this point (28). They found that when risk information is presented in a concrete numerical format, individuals tend to be more accurate in evaluating probabilities and making decisions. This format reduces cognitive load, facilitating more intuitive information processing. It is particularly effective for populations with limited understanding of mathematical concepts, such as some elderly individuals or those with lower levels of education. By using specific frequency descriptions, communicators can effectively mitigate the audience’s anxiety and confusion during the comprehension process, thus fostering more rational decision-making.

Lipkus conducted a comparative analysis of different forms of risk information presentation (29), further validating the effectiveness of frequency-based formats. He found that whether presented through text, numbers, or graphs, the use of frequency expressions (e.g., “5 out of 100 people”) helps individuals better understand the magnitude of health risks and motivates them to take appropriate preventive actions. In the context of health communication and the dissemination of medical information, frequency formats simplify the transmission of complex statistical concepts, reducing patient misunderstandings and enhancing their ability to comprehend risk information.

Combined with the doctor-patient scenario, the results of this study suggest that in doctor-patient communication, healthcare professionals should actively construct and fulfill psychological contracts and use numerical representations that fit the patient’s mental model to reduce the patient’s perceived error of medical risk. Furthermore, these results provide actionable recommendations for medical training and communication practices. On one hand, at the hospital management level, when hospitals effectively fulfill psychological contracts, doctors feel trusted, supported, and valued in their contracts with the hospital, making it easier for them to display empathy and make stable professional decisions when interacting with patients (30). In doctor-patient relationships, doctors can enhance patients’ trust by establishing psychological contracts (e.g., clearly communicating and fulfilling promises) during the communication process. Additionally, frequency formats (e.g., “5 out of 100 people”) can be used in risk communication to improve patients’ understanding of the information.

Second, for pain empathy, in the no psychological contract condition, response times to pain pictures were significantly higher in the 1-in-X form than in the other two forms, meaning that the 1-in-X form was detrimental to the participants’ ability to develop pain empathy, whereas the difference in response times to pain empathy between the X-in-100 and X% forms was not significant. Contrarily, the fulfillment of the psychological contract led to a reduction in the difference between the three forms of data representation for the production of pain empathy. The establishment of the psychological contract significantly enhanced the participants’ pain empathy. This is consistent with the previous findings (31). Recent research suggests that healthcare professionals may no longer view their profession as a mere occupation, but rather expect an acceptable work-life balance and appropriate financial rewards for their efforts (32). This shift has transformed the psychological contract between healthcare professionals and patients from a relational focus emphasizing mutual investment within long-term employment relationships to a transactional focus prioritizing clear duties and responsibilities within short-term employment arrangements (33). The shift in the form of the contract makes it possible for health care providers to focus more on the current state of expectation, with a heightened sense of inner need for fulfillment of the psychological contractual situation.

From the perspective of a short-term psychological contract, trust and commitment between patients and healthcare professionals are founded on specific medical services. For example, patients expect timely and professional diagnoses and treatments, while healthcare professionals expect patients to adhere to treatment plans. Such a short-term contract plays an important role in promptly resolving health issues and improving communication efficiency between patients and healthcare professionals, thereby enhancing patient satisfaction in the short term (34).

On the other hand, a long-term psychological contract emphasizes emotional connection and enduring commitment between patients and healthcare professionals. In a long-term patient-caregiver relationship, healthcare professionals not only address a patient’s current medical condition but also focus on long-term improvements in overall health and quality of life. Through ongoing interactions, patients gradually develop deep trust in their healthcare providers, becoming more receptive to medical advice and establishing a collaborative relationship in health management. A long-term psychological contract contributes to better patient adherence, improved overall treatment outcomes, and reduced conflicts between patients and healthcare professionals (35).

To summarize, short-term psychological contracts emphasize immediacy and professionalism in medical services, whereas long-term psychological contracts are built upon sustained trust and deep interaction. Together, they influence the patient-caregiver relationship on different levels, forming a more comprehensive and harmonious model of patient-care interaction. Ultimately, the fulfillment of psychological contracts is crucial in the healthcare context, as it enhances empathetic understanding from healthcare professionals and strengthens the overall therapeutic relationship.

In conclusion, the fulfillment of the psychological contract is undoubtedly important in the doctor-patient relationship, and can enhance the pain empathy of healthcare professionals. In situations where a psychological contract cannot be immediately established during routine doctor-patient communication, employing a numerical format consistent with psychological models can facilitate patient comprehension of surgical risks, enable informed decision-making regarding treatment options, and foster a realistic perception of their condition and treatment. This approach mitigates the uncertainty and anxiety resulting from information asymmetry. Empirical evidence suggests that when patients are able to comprehend treatment aspects more clearly through numerical representation, their anxiety levels significantly decrease, which in turn improves their willingness to cooperate with treatment and adhere to medical recommendations (36). Moreover, this strategy has the potential to enhance healthcare professionals’ empathy towards patients, allowing for a deeper understanding of the patient’s perspective.

### Conclusion

The findings of this study indicate that the establishment of a psychological contract significantly enhances healthcare professionals’ pain empathy levels and effectively reduces probability estimation bias in risk-related decision-making. Moreover, in the absence of a psychological contract, intuitive frequency formats (such as X-in-100) were found to reduce cognitive biases and improve the efficiency of information communication. From a practical perspective, the study suggests that attention should be given to the fulfillment of psychological contracts in both doctor-patient relationships and hospital management, and that frequency-based data presentation formats should be prioritized in risk communication to enhance mutual trust and decision-making quality. Future research could further investigate the application of these findings across various clinical settings and contribute to the development of more effective healthcare communication strategies.

### Limitations and future research directions

A notable limitation of the present study is its exclusive focus on the healthcare providers’ perspective, without considering the relationship between probability estimation biases in decision-making events and empathy for pain from the patients’ viewpoint. This narrow focus may hinder a comprehensive understanding of the full dynamics involved in medical decision-making. Future research should adopt a dual-perspective approach that includes both healthcare professionals and patients, facilitating a comparative analysis of their responses. This would allow for a more thorough exploration of the similarities and differences in their perceptions, ultimately leading to a deeper understanding of the doctor-patient relationship.

Furthermore, the study did not fully incorporate probability estimation biases and data formats into real-world medical-patient contexts, which may limit the generalizability of the findings to clinical decision-making scenarios. Future research should account for medical risks and specific decision-making environments to enhance the ecological validity of the results.

Finally, there is currently no standardized operational definition for psychological contracts. In this study, psychological contract manipulation was operationalized through the use of gifts, following suggestions from healthcare professionals and validated via a brief post-experiment survey to manipulation check. However, this approach does not entirely mirror real-life clinical interactions, potentially affecting the ecological validity of the findings. Future research should refine the operationalization of psychological contracts and explore alternative methods of manipulation, such as verbal commitments or written agreements, to better understand their impact on doctor-patient interactions.

## Data Availability

The datasets presented in this study can be found in online repositories. The names of the repository/repositories and accession number(s) can be found below: https://osf.io/q7fn3/files/py6wz.
