# Correction: From numerical to empathy: the dual impact of psychological contracts in doctor-patient communication

**DOI:** 10.3389/fpsyt.2025.1737146

**Published:** 2025-11-12

**Authors:** Xinru Wang, Yating Chen, Yi Yu, Huan Jiang, Jinyan Song, Weixian Liang, Qiang Zhou, Liang Ying

**Affiliations:** 1 Wenzhou Medical University, Wenzhou, China; 2 Soochow University, Suzhou, China

**Keywords:** pain, empathy, psychological contracts, doctor-patient relationship, probability estimation bias

In the published article, authors “Xinru Wang, Yating Chen, and Yi Yu” were erroneously not credited as equal contributors. The original version of this article has been updated.

